# Genomic selection for carrier-state resistance in chicken commercial lines

**DOI:** 10.1186/1753-6561-5-S4-S24

**Published:** 2011-06-03

**Authors:** Fanny Calenge, Andres Legarra, Catherine Beaumont

**Affiliations:** 1 INRA UMR83 Unité de Recherches Avicoles, 37380 Nouzilly, France; 2 INRA UR0631 Station d’Amélioration Génétique des Animaux, chemin de Borde-Rouge, 31326 Auzeville, France

## Abstract

**Background:**

*Salmonella* propagation by apparently healthy chicken and subsequent food security concerns could be decreased by the selection and use of chicken lines more resistant to carrier-state. In the present study we applied the first steps of the genomic selection methodology to assess the interest of including genetic markers for the genetic evaluation of hen lines infected with *Salmonella Enteritidis*.

**Methods:**

We studied commercial laying hen lines divergently selected for resistance to *Salmonella* carrier-state at two different ages. A total of 600 animals were typed with 1536 SNP markers and artificially infected with *S.* Enteritidis. Phenotypes were collected four weeks (young animals) or five weeks (adults) later. Two types of variance component analyses, including or not including SNP data, were performed and compared. All variance components were estimated by Bayesian methods and Gibbs sampling.

**Results:**

The comparison of both genetic analyses shows that SNP are efficient in capturing genetic variation, although none of them captures a large affect on the traits studied. Average accuracies do not change between analyses, showing that using SNP data does not really increase information.

**Conclusions:**

These preliminary results show that genomic selection for *Salmonella* carrier-state resistance in laying hens is promising, although a denser SNP coverage of the genome on a higher number of animals is needed to assess its feasibility and efficiency compared to classical pedigree evaluation.

## Background

Like most disease resistance related traits, resistance to *Salmonella* carrier-state has a rather weak heritability [1]; in addition selection has to be conducted on siblings of infected animals. Therefore selection efficiency for this trait could probably be improved by genomic selection [2]. Resistance to carrier state is the animal’s ability to rapidly eliminate *Salmonella*, once infected. The use of chicken more resistant to *Salmonella* carrier-state could be a way to decrease the propagation of *Salmonella* in poultry stocks, which could have a direct impact on food safety. The feasibility of selection for an improved resistance to carrier state has been demonstrated by a divergent selection experiment from a laying hen commercial line [1]. In the present study we applied the first steps of the genomic selection methodology [3] to assess the interest of including genetic markers for the genetic evaluation of the aforementioned lines infected with *Salmonella Enteritidis*..

## Methods

### *Salmonella* challenges

The S. Enteritidis (SE) nalidixic acid and streptomycin resistant PT 4 strain 1009 was used for all challenges. Chicks and adult hen resistance was assessed as described previously [4,5]. Three hundreds and eighty nine chicks were orally inoculated. Caeca bacterial counts were expressed in log (cfu) per gram of caeca (trait Young_Caeca_). Two hundreds and eight hens were inoculated at the peak of lay The presence/absence of *Salmonella* in spleen (Adult_Spleen_), liver (Adult_Liver_) and caeca (Adult_Caeca_) and the global contamination rate Adult_0-1_ (0 for no organ contaminated/ 1 for one or more organ contaminated) were considered for further analyses.

### SNP markers obtention

SNP marker genotypes were obtained by the Illumina Golden Gate technology on a BeadExpress station (KOS Genetic, Italy); 194 markers were chosen to specifically cover three previously identified QTL regions on chromosomes 1, 2 and 5 [6,7], while the remaining 1342 SNP markers were chosen to cover homogeneously the entire genome.

### Genetic evaluation

For each of the 5 traits, two preliminary analyses were performed. Approach PEDVC (pedigree-variance components): regular estimation of genetic parameters using the usual pedigree-based relationship matrix **A**. Approach COMVC (combined variance components): two random effects, one with covariance matrix **A** (thus based on pedigree) and the other using a combined pedigree-genomic relationship matrix **H**[8]. After variance component estimation, and using a point estimate of variance components, BLUP [9] estimates of genetic values were computed using either the matrix **A** only (PEDBLUP), or including the pedigree-genomic relationship matrix **H** (COMBLUP) as well. To obtain only one EBV a new relationship matrix was created weighting each matrix by its associated variance component [10]. The theoretical accuracy *r* was computed in both cases from the diagonal elements (PEV) of the inverse of the mixed model equations, as , where  is the genetic variance. In both variance components and BLUP estimates, we used the BLUPF90 series of programs http://nce.ads.uga.edu/~ignacy/newprograms.html, with the modifications included to account for genomic relationship matrices [11].

## Results

### SNP markers obtention

From 1536 original SNP, only 831 turned out to be polymorphic; the rest were discarded from statistical analyses. A set of 141 SNP were not in Hardy-Weinberg equilibrium (p<10^-6^), but they were retained in the analysis, because a selected population is not expected to behave in Hardy-Weinberg equilibrium [12].

### Genetic evaluation

Table 1 shows estimates of genetic parameters. For most traits, standard errors of heritability are around 0.07. On the one hand, estimated residual variances do not change between analyses. This implies that none of the SNP captures a large effect on the trait, despite some being in QTL regions. On the other hand, it can be seen that in the second analysis (Combined) most (but not all) heritability is captured by markers. Indeed, heritability explained by markers ranges between 40% and 90% of all heritability. This shows that markers are efficient in capturing genetic information.

**Table 1:** Estimates of genetic parameters of analysis with pedigree only (PEDVC) or pedigree and SNP markers (COMVC)

Analysis		Young_log(cfu)_	Adult_liver_	Adult_Spleen_	Adult_Caeca_	Adult_0-1_
PEDVC	Var(e)	1.72	0.011	0.022	0.17	0.18
	h^2^_u_	0.17	0.04	0.17	0.18	0.21
COMVC	Var(e)	1.85	0.011	0.024	0.18	0.19
	h^2^_u_	0.048	0.002	0.019	0.02	0.02
	h^2^_h_	0.034	0.009	0.079	0.13	0.19

Residual variance and heritability explained by pedigree (h^2^_u_) or markers (h^2^_h_)

Table 2 and Figure 1 show theoretical accuracies computed from the inverse of the matrix of mixed model equations in each model. Average accuracies do not change, which confirms that the use of SNP did not really increase the information. However, for adult measures, their standard deviation increases, particularly for liver contamination.

**Table 2:** Mean ± standard deviation of theoretical accuracies from mixed model equations using either pedigree only (PEDVC) or pedigree and SNP markers (COMVC)

	Young_log(cfu)_	Adult_liver_	Adult_Spleen_	Adult_Caeca_	Adult_0-1_
PEDVC	0.41 ±0.02	0.08 ±0.03	0.45 ±0.04	0.52 ±0.04	0.59 ±0.04
COMVC	0.42 ±0.05	0.20 ±0.09	0.42 ±0.08	0.51 ±0.06	0.59 ±0.05

## Conclusions

Overall, the use of SNP does not change the picture of genetic evaluation. Genetic parameters and theoretical accuracies are similar. This is probably partly due to an insufficient SNP density and to a lack of phenotypic data. A denser SNP coverage and more phenotypic data are thus needed to perform a more efficient evaluation of the interest of including SNP markers for the genetic evaluation of hens infected with *S.* Enteritidis, which is the first step toward genomic selection.

## Competing interests

The authors declare they have no competing interest.

## Authors' contributions

AL performed the genetic evaluations. FC and CB acquired phenotypic and genotypic data. AL and FC drafted the manuscript.
